# Attention deficit and hyperactivity disorder: a therapeutic option

**DOI:** 10.1590/S1679-45082014AO2925

**Published:** 2014

**Authors:** Abram Topczewski

**Affiliations:** 1 Hospital Israelita Albert Einstein, São Paulo, SP, Brazil.

**Keywords:** Attention deficit disorder with hyperactivity, Antidepressants/therapeutic use, Anxiolytics/therapeutic use, Central nervous system stimulants

## Abstract

**Objective:**

To evaluate the use of a therapeutic regimen to treat attention deficit hyperactivity disorder patients.

**Methods:**

A total of 140 patients initially underwent physical, neurological and laboratory evaluation. Thereafter, treatment was initiated with a compounding product consisting of a tricyclic antidepressant and an anxiolytic.

**Results:**

The response was positive in 71.43% of patients in controlling hyperactivity and improving dispersion and attention deficit.

**Conclusion:**

The therapeutic regimen utilized proved to be an effective therapeutic alternative, especially for patients who do not adapt to psychostimulant drugs.

## INTRODUCTION

Attention deficit hyperactivity disorder (ADHD) is a clinical condition very prevalent in childhood and adolescence. The mean global prevalence in children is estimated to be 5.3%.^(^^1^^)^ It predominates in males, at a 3:1 or 2:1 rate, depending on the author. It features attention deficit, hyperactivity, and impulsiveness. The clinical manifestations may present as combined symptoms; or either attention deficit or hyperactivity-impulsiveness predominating. It is a neurobiological dysfunction, resulting from genetic factors but with important environmental influences.

Amongst the environmental factors responsible for ADHD we must mention tobacco, alcohol, and cocaine use during pregnancy; and fetal distress, low birth weight and prematurity in the perinatal and postnatal periods.^(2)^ The genetic-environment combination is the determinant factor of the clinical diversity manifested by ADHD patients; beyond that, one must also consider the comorbidities that many patients present, such as oppositional defiant disorder, behavioral disorder, anxiety disorder, depression, obsessive-compulsive disorder (OCD), tics, migraine, enuresis, encopresis, dyslexia, epilepsy, and use of illicit substances.^(3,4)^ ADHD in itself causes much impact on performance at school, family relationship, and social life. When together with any of the above-mentioned comorbidities, it achieves a much larger dimension and sometimes is very concerning. School performance beyond the intellectual capacity of the child is an extremely important matter, since it brings much emotional suffering, affecting self-esteem and self-concept. Currently, the treatment advocated for ADHD is use of psychostimulants,^(5)^ but not all patients adapt well to the use of those medications due to side effects. In the current study a treatment modality for ADHD was presented, associating a tricyclic antidepressant and an anxiolytic.

## OBJECTIVE

To retrospectively evaluate a series of patients with attention deficit hyperactivity disorder treated with tricyclic antidepressant associated to an anxiolytic.

## METHODS

No patient of the sample was on treatment for less than 6 months. All were neurologically examined by the same person, in a clinic, and followed up during treatment. At the beginning of the treatment, patients’ complaints (when pertinent), information provided by relatives, school reports, and the assessments made available by professionals engaged in the management (speech therapist, psychologist, psychopedagogist and teacher) were considered. The patients of the sample were already being cared for by those professionals, since they had been previously referred by the school councils. The patients came to the office as suggested by the attending professionals because the results achieved with treatment were beyond expectations. Before initiating drug therapy, all patients underwent laboratory tests, electroencephalography and electrocardiography. The clinical-neurological evaluations were performed every 2 months, and laboratory tests repeated every 5 to 6 months. Treatment efficacy was evaluated based on information provided by patients, reports of parents, school evaluations, and by the reports by practitioners engaged in the treatment.

The treatment proposed was a combination of imipramine and chlordiazepoxide, compounded in specialized pharmacies, since it is not available in the market and the components could be adjusted to each individual. The treatment initially advocated was a combination of imipramine (1.2mg/kg/day) and chlordiazepoxide (0.05-0.08mg/kg/day), single dose at dinner. The maximum daily doses of imipramine and chlordiazepoxide were 40mg and 3.5mg, respectively.

The study was approved by the Research Ethics Committee of the *Hospital Israelita Albert Einstein* (CEP 920, CAAE: 0145002800008).

## RESULTS

The therapeutic objectives related to improving hyperactivity, attention deficit, anxiety, and sleeping pattern were positive in 100 (71.43%) patients in our group. In 40 (28.57%) patients, the results were partial in control of hyperactivity and/or attention deficit. The group that did not had the expected response in the behavior, academic or social aspects, received other medications, such as psychostimulants (methylphenidate, lisdexamphetamine and modafinil), atypical neuroleptic (risperidone), or other type of antidepressive drug (bupropion). Most patients complaining of sleep disorders, such as going to bed late, difficulty in falling asleep, agitated sleep, or who complained of waking at night, benefited much from the regimen suggested. This improvement in sleeping pattern, which was quite relevant, may be related to the sedative effect of chlordiazepoxide, besides the improvement in anxiety and control of hyperactivity. Family members referred that sleep became calmer and that they woke up earlier than before, but more rested and active. Improvement in anxiety and voracity were quite evident. Some adverse events were reported, such as appetite reduction, dry mouth, intestinal constipation, abdominal pain, headache, dizziness, and palpitation. The majority of these manifestations was mentioned by 28 (20%) of the patients in the initial phase of the treatment, considered the period of adaptation to the medication. None of the patients of the sample presented relevant side effects as to justify withdrawing the treatment.

## DISCUSSION

Although the majority of authors considers that the initial treatment proposed for ADHD should be psychostimulants,^(5,7,8)^ tricyclic antidepressants represent an interesting and efficient alternative,^(9)^ since they may benefit patients consistently, especially when there are comorbidities. According to Barkley,^(10)^ one third of ADHD patients do not adapt to the use of psychostimulants. Since the medications proposed in this sample (imipramine and chlordiazepoxide) were compounded, this allowed for individual tailoring of the dose, minimizing adverse effects and rendering positive results in treating ADHD symptoms, as well as the associated conditions. The financial issue should also be mentioned, because compounded medications are much cheaper than psychostimulants. In Brazil, where low socioeconomic status predominates, tricyclic antidepressants represent an important alternative, since the social reach is much larger. Those medications have a beneficial effect on mood, anxiety, tics, voracity, and sleep disorders. There is some concern about the use of benzodiazepines as to drug abuse and dependence.^(11)^ This is true but only for the most recent generation benzodiazepines. The possibility of dependence or the excessive uncontrolled use of this compound (imipramine and chlordiazepoxide) is remote, since it does not encompass potential for abuse.^(12)^ The same is not true for psychostimulants, which have been prescribed and consumed in an increasingly growing manner.^(13)^ In this sample we present part of an experience of treating those patients with tricyclic antidepressants associated, in their formulation, to an anxiolytic, for many years. This combination proved to be very efficient, considering improvement in behavior, hyperactivity, and as a consequence, better family relationship and interactions outside the family. The attention and concentration levels also improved, leading to marked improvement in performance at school. This sum of benefits dramatically changed self-esteem and self-concept, which were negatively affected. Tricyclic antidepressants, due to a continuous use during three to four years modify brain metabolism, improve functional aspects, and, associated to the process of brain maturation, tend to promote definitive improvement in ADHD; hence medications can be discontinued not affecting family, social or school conditions. ADHD patients had signs of brain hypoperfusion (frontal and parietal regions, and striatum) on a single-photon emission computerized tomography (SPECT) upon diagnosis, and on clinical discharge, the perfusion disturbances were no longer detected on SPECT (author’s communication). In addition, those medications benefit patients when there are some comorbidities, such as OCD, tics, Tourette syndrome, depressive state, panic, enuresis, and encopresis.^(2,6)^ The association of tricyclic antidepressants to benzodiazepines proved efficient in patients with sleep disorders, such as agitated sleep, frequent awakening, somniloquy, and excessive movements in bed. The poor night sleep hinders waking up in the morning, interferes in performance at school due to tiredness, and sometimes causes daytime sleepiness.^(14)^

This tiredness renders the child irritated and intolerant, besides disturbing the attention and concentration states. In a retrospective evaluation, we verified that a relevant number of children with ADHD presented changes in sleep rhythm since infancy. Feeding disorders play a relevant role in ADHD patients, especially binge eating, voracity and obesity. Such disturbances come in a close relation to the state of anxiety, and in those cases, benzodiazepines are quite efficient. The tricyclic antidepressants may be given once a day, since the effects are sustained for 24 hours, and have a lasting action over attention deficit and hyperactivity. They do not cause anxiety, tics, or feeding compulsion at the end of the day, as observed with psychostimulants. Furthermore, there is the advantage of not causing anorexia, with important weight loss. Some patients presented increased appetite but with no significant changes in body mass. The ADHD symptoms result from dysfunction of brain neurotransmitters, particularly dopamine, noradrenaline and serotonin. Tricyclic antidepressants act blocking serotonin and norepinephrine reuptake, their action over dopamine being much more fragile.^(15)^ The adrenergic pathways exert important action in maintaining attention, concentration, and motivation. Anxiety seems to be related to hyperactivity of the adrenergic system or to a serotonergic dysfunction. Serotonin depletion interferes in the sleep mechanism, and the subsequent disturbances may be manifested in many ADHD patients.

The aim of treatment is the behavioral improvement related to hyperactivity, impulsiveness, intolerance to frustration, and aggressiveness. Behavior improvement will have important impact in the relationship with relatives, teachers, friends, and schoolmates. Additionally, the treatment has favorable effects for the attention and concentration levels, leading to better memorization, what renders learning more efficient. The sum of improvements helps to correct self-concept and self-esteem distortions, putting at balance the emotional state. In addition to the individualized pharmaceutical treatment, in some cases psychoeducation and family counseling should be associated.^(16)^

## CONCLUSION

The treatment proposed proved to be an efficient alternative for attention deficit disorder and hyperactivity patients who do not adapt to psychostimulants.
