# Supplementary material for: Attention deficit and hyperactivity disorder: a therapeutic option
Source: Einstein (Sao Paulo). 2014 Jul-Sep;12(3):310–3. doi: 10.1590/S1679-45082014AO2925 (PMC4872941; doi:10.1590/S1679-45082014AO2925)
Supplement: Translation

# Translation: Transtorno do déficit de atenção e hiperatividade: uma vertente terapêutica

**Authors:** Abram Topczewski

**Affiliations:** 1 Hospital Israelita Albert Einstein, São Paulo, SP, Brasil.

**Keywords:** Transtorno do déficit de atenção com hiperatividade, Antidepressivos/uso terapêutico, Ansiolíticos/uso terapêutico, Estimulantes do sistema nervoso central

## Abstract

**Objetivo:**

Avaliar a aplicação de um esquema terapêutico para o tratamento do transtorno do déficit de atenção e hiperatividade.

**Métodos:**

Os 140 pacientes foram submetidos inicialmente à avaliação clínico-neurológica e laboratorial. Posteriormente, foi iniciado um tratamento com medicamento formulado composto por um antidepressivo tricíclico e um ansiolítico.

**Resultados:**

A resposta foi positiva em 71,43% dos pacientes no controle da hiperatividade e na melhoria do quadro de dispersão e desatenção.

**Conclusão:**

O esquema terapêutico aplicado se mostrou uma alternativa terapêutica eficaz, especialmente para os pacientes que não se adaptam aos medicamentos psicoestimulantes.

## INTRODUÇÃO

O transtorno do déficit de atenção e hiperatividade (TDAH) é um quadro clínico bastante prevalente na infância e na adolescência. Estima-se que a média mundial, na criança, seja de 5,3%.^(^^1^^)^ É predominante no gênero masculino, na razão de 3:1 ou 2:1, dependendo do autor. Caracteriza-se por déficit de atenção, hiperatividade e impulsividade. As manifestações clínicas podem se apresentar na forma combinada dos sintomas; na forma em que predomina o déficit de atenção; ou na qual predomina a hiperatividade-impulsividade. Trata-se de uma disfunção neurobiológica, que se manifesta em consequência de fatores genéticos, mas com importantes influências ambientais.

Dentre os fatores ambientais responsáveis pelo TDAH, devemos citar o consumo de tabaco, álcool e cocaína no período gestacional; nos períodos peri e pós-natal, mencionam-se o sofrimento fetal, o baixo peso e a prematuridade.^(2)^ A conjugação genética-ambiental é o fator determinante da diversidade clínica manifestada pelos pacientes portadores do TDAH; além disso, devemos considerar as comorbidades que muitos pacientes apresentam, como o transtorno desafiador opositor, transtorno de conduta, transtorno de ansiedade, depressão, transtorno obsessivo-compulsivo (TOC), tiques, migrânea, enurese, encoprese, dislexia, epilepsia e consumo de substâncias ilícitas.^(3,4)^ Grande é o impacto que o TDAH causa, por si só, no tocante ao rendimento escolar, ao relacionamento familiar e à vida social do indivíduo. Quando acompanhado de alguma das comorbidades referidas, o TDAH alcança uma dimensão muito maior e, em algumas ocasiões, torna-se bastante preocupante. O desempenho escolar aquém da capacidade intelectual da criança é uma questão extremamente importante, pois promove grande sofrimento emocional, comprometendo a autoestima e autoimagem. Atualmente, o tratamento preconizado para o TDAH é realizado com psicoestimulantes,^(5)^ porém nem todos os pacientes se adaptam bem ao uso desses medicamentos, por conta dos efeitos adversos. No presente estudo, foi apresentada uma modalidade terapêutica do TDAH, em que associaram medicamento antidepressivo tricíclico e um ansiolítico.

## OBJETIVO

Avaliar retrospectivamente uma série de pacientes portadores de transtorno de déficit de atenção e hiperatividade tratados com medicamento antidepressivo tricíclico associado a ansiolítico.

## MÉTODOS

Nenhum dos pacientes da amostra estava em tratamento por tempo inferior a 6 meses. Todos foram submetidos a exame clínico neurológico pelo mesmo examinador, em uma clínica, e acompanhados durante o tratamento. Ao início do tratamento, foram consideradas as queixas apresentadas pelos pacientes (quando passíveis de serem consideradas), os relatos dos familiares, os relatórios fornecidos pelas escolas e as avaliações disponibilizadas pelos profissionais envolvidos no tratamento (fonoaudiólogo, psicólogo, psicopedagogo e professor). Os pacientes da amostra já se encontravam sob orientação dos profissionais, pois haviam sido encaminhados, previamente, pelos orientadores da escola. Os pacientes vieram ao consultório por sugestão dos profissionais que os atendiam, pois os resultados alcançados somente com as terapias em curso estavam a desejar. Antes de ser iniciado o tratamento medicamentoso, todos os pacientes foram submetidos a exames laboratoriais, especialmente o eletroencefalograma e o eletrocardiograma. As avaliações clínico-neurológicas eram realizadas a cada 2 meses, e os exames laboratoriais repetidos a cada 5 a 6 meses. A eficácia terapêutica foi considerada pelas informações fornecidas pelos pacientes, pelos relatos dos pais, pelas avaliações escolares e pelos relatórios dos profissionais envolvidos no tratamento.

O tratamento proposto foi um composto de imipramina e clordiazepóxido, que era manipulado em farmácias especializadas, por não estar disponível no mercado e para que fosse possível a adaptação individual dos componentes. O tratamento inicialmente preconizado foi a composição de imipramina (1,2mg/kg/dia) e clordiazepóxido (0,05-0,08mg/kg/dia), em dose única, ao jantar. As doses máximas diárias ministradas de imipramina e clordiazepóxido foram 40mg e 3,5mg, respectivamente.

O artigo foi aprovado pelo Comitê de Ética em Pesquisa do Hospital Israelita Albert Einstein (CEP 920, CAAE: 0145002800008).

## RESULTADOS

Os objetivos terapêuticos relacionados à melhoria de hiperatividade, déficit de atenção, ansiedade e padrão do sono foram positivos em 100 (71,43%) dos pacientes do nosso grupo. Em 40 (28,57%) pacientes, os resultados foram parciais no controle da hiperatividade e/ou do déficit de atenção. Ao grupo que não apresentou a resposta esperada, do aspecto comportamental, acadêmico ou social, foram ministrados outros medicamentos, como psicoestimulante (metilfenidato, lisdexanfetamina e modafinil), neuroléptico atípico (risperidona), ou outro tipo de antidepressivo (bupropiona). A maioria dos pacientes que referia alterações do sono, como ir dormir tarde, dificuldade para conciliar o sono, sono agitado, ou que se queixava de despertares noturnos, muito se beneficiou com o esquema terapêutico formulado. Essa melhoria do padrão do sono, que foi bastante relevante, pode estar relacionada ao efeito sedativo do clordiazepóxido, além da melhoria do estado ansioso e ao controle da hiperatividade. Os familiares referiam que o sono se tornara mais tranquilo e que eles despertavam mais cedo que anteriormente, porém mais descansados e dispostos. A melhoria do estado ansioso e da voracidade alimentar foi muito evidente. Alguns efeitos adversos foram relatados, como redução do apetite, boca seca, obstipação intestinal, dor abdominal, cefaleia, tonturas e palpitação. A maior parte dessas manifestações foi referida por 28 (20%) pacientes na fase inicial do tratamento, considerado o período de adaptação aos medicamentos. Nenhum dos pacientes da amostra apresentou efeitos colaterais relevantes a ponto de justificar a suspensão do tratamento em curso.

## DISCUSSÃO

Embora considerado pela maioria dos autores que o tratamento inicial proposto para o TDAH deva ser feito com os psicoestimulantes,^(5,7,8)^ os antidepressivos tricíclicos representam uma alternativa interessante e eficaz,^(9)^ pois encerram bastante benefícios a esses pacientes, especialmente quando há comorbidades. Segundo Barkley,^(10)^ um terço dos pacientes portadores de TDAH não se adapta ao uso dos psicoestimulantes. Como os medicamentos propostos nessa amostra (imipramina e clordiazepóxido) eram manipulados, isso permitiu que a dose fosse adaptada individualmente, minimizando os efeitos adversos e propiciando resultados positivos no tratamento dos sintomas do TDAH, bem como das comorbidades. Outro ponto que deve ser mencionado se relaciona à questão financeira, pois os medicamentos manipulados são muito mais baratos que os psicoestimulantes. No nosso país, onde predominam as classes socioeconômicas menos favorecidas, os antidepressivos tricíclicos representam uma alternativa terapêutica importante, já que a abrangência social é muito maior. Esses medicamentos promovem efeitos benéficos sobre o humor, a ansiedade, os tiques, a voracidade alimentar e as alterações do sono. Há certa preocupação quanto ao uso do benzodiazepínicos, com relação ao abuso e à dependência da droga.^(11)^ Isso, de fato, existe, porém é verdadeiro para os benzodiazepínicos de geração mais recente. A possibilidade de dependência ou uso excessivo e descontrolado desse composto (imipramina e clordiazepóxido) é remota, pois ele não encerra potencial de abuso.^(12)^ O mesmo não se verifica com os psicoestimulantes, que são prescritos e consumidos de modo progressivamente elevado.^(13)^ Apresentamos, nesta amostra, parte de uma experiência vivenciada por vários anos de tratamento desses pacientes com antidepressivos tricíclicos associados, em sua formulação, a medicamento ansiolítico. Esse esquema mostrou-se bastante eficiente, considerando-se a melhoria do quadro comportamental, a hiperatividade e, consequentemente, a melhoria nas relações familiares, bem como as extrafamiliares. O nível de atenção e concentração também foi melhorado e isso propiciou considerável incremento do rendimento escolar. Essa somatória de benefícios alterou muito a autoestima e a autoimagem, que se encontravam negativamente comprometidas. Os antidepressivos tricíclicos, por seu uso contínuo durante um período de três a quatro anos, modificam o metabolismo cerebral, melhoram o quadro funcional e, associados ao processo de maturação cerebral, tendem a promover a melhoria definitiva do quadro do TDAH, o que permite a suspensão dos medicamentos sem qualquer comprometimento, seja na esfera familiar, social ou acadêmica. Pacientes portadores de TDAH submetidos a exame pela tomografia computadorizada por emissão de fóton único (SPECT), por ocasião do diagnóstico, revelaram sinais de hipoperfusão cerebral (região frontal, parietal, estriatal); por ocasião da alta clínica, as alterações perfusionais não foram mais detectadas pelo SPECT (comunicação do autor). Além disso, esses medicamentos apresentam vantagens em seu uso quando existem comorbidades como TOC, tiques, síndrome de Tourette, estado depressivo, pânico, enurese e encoprese.^(2,6)^ A associação dos antidepressivos tricíclicos com benzodiazepínico mostrou ser eficaz nos pacientes que apresentavam distúrbios do sono, como sono agitado, despertar noturno frequente, sonilóquio e movimentação excessiva no leito. As noites mal dormidas dificultam o despertar pela manhã bem como interferem no bom desempenho escolar, por conta do cansaço e, por vezes da sonolência diurna.^(14)^

Esse cansaço torna a criança irritada e intolerante, além de atrapalhar o estado de atenção e concentração. Em avaliação retrospectiva, verificamos que um número relevantes de crianças com TDAH já apresentou alterações do ritmo de sono desde o período lactente. Os distúrbios alimentares ocupam um lugar relevante nos pacientes com o quadro do TDAH, especialmente o comer compulsivo, a voracidade e a obesidade. Essas alterações guardam uma relação estreita com o estado de ansiedade e, nesses casos, os benzodiazepínicos são bastante eficazes. Os antidepressivos tricíclicos podem ser ministrados em dose única, pois os efeitos persistem durante 24 horas, e exercem uma ação mais duradoura sobre o défice de atenção e a hiperatividade. Não desencadeiam ansiedade, tiques e nem promovem a compulsão alimentar no final do dia, como acontece, em certas ocasiões, com os psicoestimulantes. Além disso, há a vantagem de não provocarem anorexia, com substancial perda de peso. Alguns pacientes apresentaram aumento do apetite, mas sem comprometer, de modo relevante, a massa corporal. Os sintomas encontrados no quadro do TDAH são consequentes à disfunção dos neurotransmissores cerebrais, sendo os principais a dopamina, a noradrenalina e a serotonina. Os antidepressivos tricíclicos agem bloqueando a recaptação da serotonina e da noradrenalina, sendo que sua ação sobre a dopamina é bem mais frágil.^(15)^ As vias adrenérgicas exercem importante ação na manutenção da atenção, concentração e motivação. A ansiedade parece estar relacionada a uma hiperatividade do sistema adrenérgico ou a uma disfunção serotoninérgica. A depleção da serotonina interfere no mecanismo do sono, e os seus consequentes distúrbios podem se manifestar em muitos pacientes com TDAH.

O que se pretende com o tratamento é a melhoria comportamental da hiperatividade, da impulsividade, da intolerância às frustrações e da agressividade. A melhoria comportamental terá um impacto importante nas relações com familiares, professores, amigos e colegas da escola. Além disso, o tratamento tem efeitos favoráveis para os níveis de atenção e concentração, melhorando consequentemente a memorização, o que torna o aprendizado mais eficiente. A somatória dessas melhorias concorre para corrigir as distorções da autoimagem e da autoestima, equilibrando o estado emocional do indivíduo. Além do tratamento farmacológico individualizado, em certos casos, devem-se associar a psicoeducação^(16)^ e a orientação familiar.

## CONCLUSÃO

O esquema de tratamento proposto se mostrou uma alternativa eficaz para os pacientes portadores de transtorno do déficit de atenção e hiperatividade que não se adaptam aos medicamentos psicoestimulantes.
